# Geographical variation of *Ceracris kiangsu* gut microbiota and its association with environmental factors

**DOI:** 10.3389/fmicb.2026.1752887

**Published:** 2026-03-20

**Authors:** Hong-Yu Liao, Ye-Song Ren, Jing Li, Hai-Tao Huang, Ze-Ling Li, Yang Zeng, Dao-Hong Zhu

**Affiliations:** 1 College of Life Science and Technology, Central South University of Forestry and Technology, Changsha, China; 2 College of Agriculture and Biotechnology, Hunan University of Humanities, Science and Technology, Loudi, China; 3 Key Laboratory of Development, Utilization, Quality and Safety Control of Characteristic Agricultural Resources in Central Hunan Province, Loudi, Hunan, China

**Keywords:** 16S rRNA, *Ceracris kiangsu*, geographical populations, gut microbiota, microbial community structure

## Abstract

The yellow-spined bamboo locust (*Ceracris kiangsu*) is one of the most destructive forest pests in China, causing severe damage to bamboo forests across multiple provinces. Understanding the relationship between its gut microbiota and environmental factors is crucial for revealing its ecological adaptability and migration potential. This study used 16S rRNA high-throughput sequencing to analyze gut bacterial communities of *C. kiangsu* from six geographically distinct populations in China. The results showed that Firmicutes and Proteobacteria were the dominant phyla, while bacterial diversity and composition varied significantly among populations. Precipitation and sunshine duration were identified as the main environmental factors most strongly associated with microbial community structure. These findings suggest that environmental conditions are strongly associated with variation in the gut microbiota of *C. kiangsu*, potentially affecting its adaptability and outbreak dynamics. This research provides new insights into the ecological mechanisms underlying pest distribution and offers a microbiome-based foundation for developing sustainable control strategies to reduce the agricultural and forestry losses caused by this species.

## 1 Introduction

Insects are among the most diverse and widely distributed organisms on Earth, inhabiting almost every terrestrial and freshwater ecosystem. Their successful colonization across diverse environments is largely attributed to their remarkable physiological plasticity and evolutionary adaptability, which often manifest as distinct geographic variations in morphology, behavior, and physiology. Such geographic differentiation enables insect species to optimize their survival and reproduction under local environmental conditions (Dillon and Lozier, 2019).

Geographical variation in insects has been extensively documented in traits such as body size, developmental duration, diapause intensity, and cold tolerance, which are shaped by local climatic factors including temperature, precipitation, and photoperiod. For instance, high-latitude populations of *Velarifictorus micado* exhibit shorter larval development and lower proportions of macropterous individuals than those in low latitudes (Zeng and Zhu, 2014), while the migratory locust *Locusta migratoria* shows increased egg cold hardiness in northern populations compared with southern ones (Jing and Kang, 2003; Tanaka and Zhu, 2008). These patterns highlight the crucial role of geographic and ecological factors in driving local adaptation.

In recent years, the insect gut microbiota has emerged as a key component influencing host physiology, nutrition, immunity, and adaptation to environmental stressors. The gut microbial community interacts closely with the host, forming a dynamic symbiotic system that contributes to digestion, detoxification, and defense against pathogens. Increasing evidence indicates that gut microbiota composition is affected by both intrinsic host factors (species, developmental stage, diet) and extrinsic environmental variables (temperature, humidity, altitude, and light intensity) (Zhang et al., 2023; Liu et al., 2019; Kang et al., 2020). Furthermore, geographic variation in gut microbiota has been reported in multiple insect taxa, such as *Holotrichia parallela* (Huang and Zhang, 2013), *Leptinotarsa decemlineata* (Yu Y. et al., 2021), *Cephalcia chuxiongica* (Yu H. et al., 2021), and *Laodelphax striatellus* (Duan et al., 2020), suggesting that microbial diversity may contribute to local adaptation mechanisms.

The yellow-spined bamboo locust (*Ceracris kiangsu*) belongs to Acrididae: Ceracrinae and is widely distributed across southern and central China, including Guangdong, Guangxi, Hunan, Hubei, Sichuan, and Yunnan provinces (Liu et al., 2021). It is one of China’s most destructive forest pests, causing severe damage to economically important bamboo species such as *Phyllostachys heterocycla* and *Bambusa textilis* (Li et al., 2018; Fan et al., 2014). Outbreaks of *C. kiangsu* can lead to widespread defoliation, reduced bamboo growth, and significant economic losses. For example, the 2020 invasion into Yunnan, China infested > 10,000 ha of agroforestry land, while historical records reported ∼6,000 ha infested in Hunan, China in 1946 (Li et al., 2025; Fan et al., 2014). Previous research has focused mainly on its ecology and population dynamics, yet little is known about the role of its gut microbiota in adaptation and dispersal across different ecological regions.

Given the ecological and economic importance of *C. kiangsu*, understanding the geographic variation in its gut microbial communities is essential for elucidating how environmental factors shape insect–microbe symbioses. In this study, we analyzed the gut microbiota of *C. kiangsu* populations from six geographically distinct regions in China (Yunnan, Guangdong, Guangxi, Hunan, and Hubei) using 16S rRNA high-throughput sequencing. We aimed to (1) characterize the gut bacterial diversity and community composition across geographic populations; (2) examine the influence of environmental factors on microbial diversity; and (3) explore potential links between gut microbiota variation and host ecological adaptation. These findings will contribute to a better understanding of the ecological adaptability of *C. kiangsu* and provide theoretical support for the development of microbe-based pest management strategies.

## 2 Materials and methods

### 2.1 Sample collection

**FIGURE 1:**
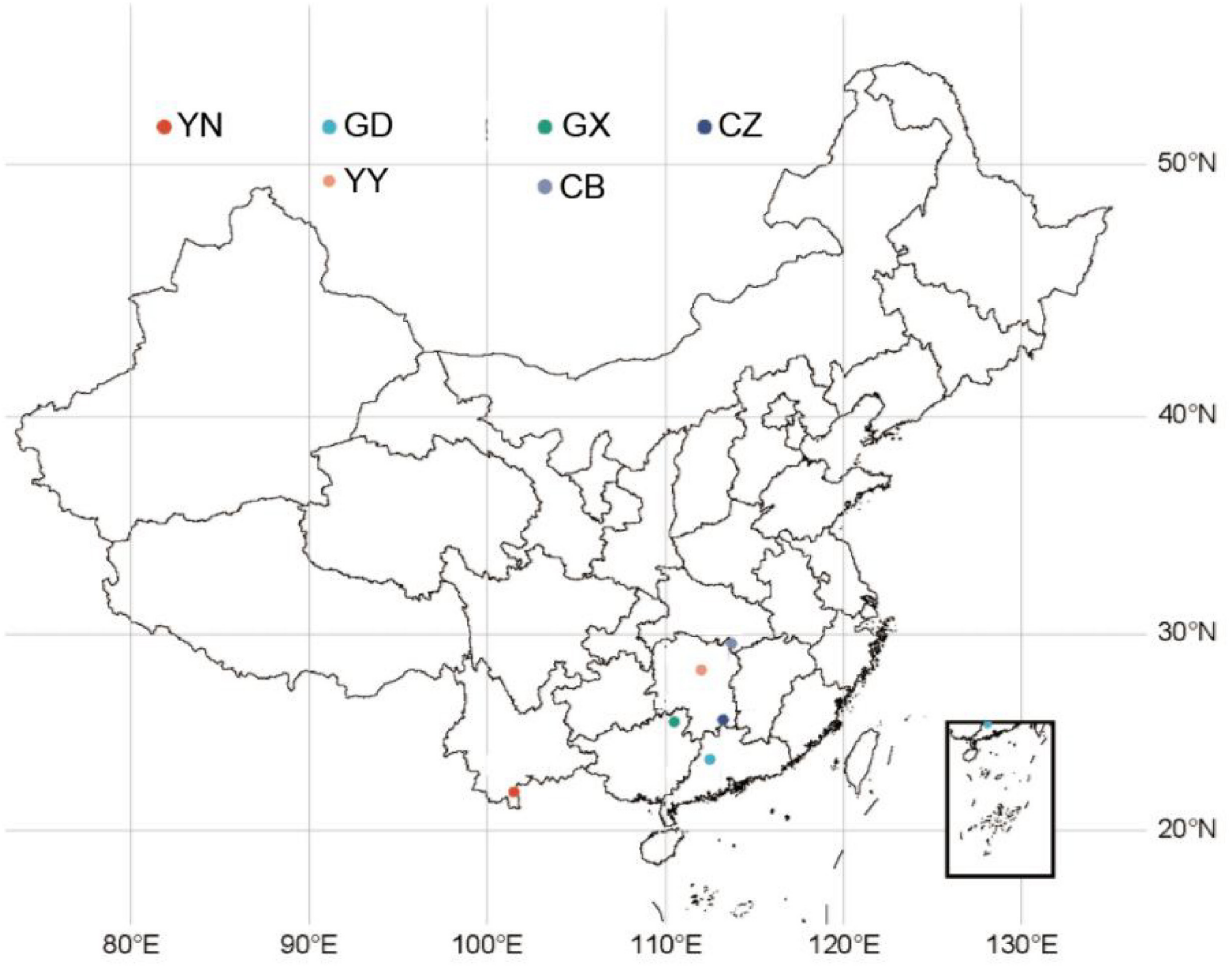
Sampling sites of *C. kiangsu*. YN, Mengla (Yunnan); GD, Guangning (Guangdong); GX, Xing’an (Guangxi); CZ, Suxian (Chenzhou, Hunan); YY, Taojiang (Yiyang, Hunan); CB, Chibi (Hubei).

The Adult female *C. kiangsu* were collected in July and August 2022 from six locations across China: Mengla, Xishuangbanna, Yunnan Province (101.5°N, 22.03°E) (YN); Guangning, Zhaoqing, Guangdong Province (112.5°N, 23.73°E) (GD); Xing’an, Guilin, Guangxi Autonomous Region (110.49°N, 25.64°E) (GX); Suxian District, Chenzhou, Hunan Province (113.24°N, 25.75°E) (CZ); Taojiang, Yiyang, Hunan Province (112.01°N, 28.25°E) (YY); and Chibi, Xianning, Hubei Province (113.71°N, 29.55°E) (CB). After collection, individuals from each site were maintained separately in clean rearing cages and starved for 48 h to allow defecation and reduce gut contents prior to dissection, following established grasshopper gut-microbiome sampling practices. Each sample consisted of three adult females, with five biological replicates per site (6 sites × 5 replicates × 3 individuals per replicate = 90 total individuals, representing 30 pooled samples). Before dissection, each insect was surface-sterilized by immersion in 70% ethanol for 5 min and rinsed 2–3 times with sterile 1 × PBS. Dissections were performed in a laminar-flow cabinet on sterilized glass slides using sterilized instruments. The entire gut was aseptically removed, and gut tissues were gently rinsed with pre-chilled sterile 1 × PBS to remove residual lumen contents and loosely associated microbes before being transferred to sterile tubes on ice. Gut tissues were then stored at –80°C (Wang et al., 2020; Bai et al., 2022; Tutagata et al., 2024) until further analysis. Sampling locations are shown in Figure 1. rr

### 2.2 DNA extraction and sequencing

Under sterile conditions, entire gut tissues were dissected from *Ceracris kiangsu* specimens using sterilized instruments and homogenized with a tissue grinder. The V3–V4 hypervariable regions of the 16S rRNA gene were amplified using the primers 338F (5’-ACTCCTACGGGAGGCAGCAG-3’) and 806R (5’-GGACTACHVGGGTWTCTAAT-3’) (Huang et al., 2021), each carrying a unique barcode sequence. PCR reactions were performed in a 20 μL volume containing 4 μL of 5 × TransStart FastPfu buffer, 2 μL of 2.5 mM dNTPs, 0.8 μL of each primer (5 μM), 0.4 μL of TransStart FastPfu DNA polymerase, and 10 ng of template DNA. The thermal cycling protocol consisted of an initial denaturation at 95°C for 3 min, followed by 27 cycles of 95°C for 30 s, 55°C for 30 s, and 72°C for 30 s, with a final extension at 72°C for 10 min. PCRs were performed in triplicate for each sample. Amplicons from the same sample were pooled and purified from 2% agarose gels using the AxyPrep DNA Gel Extraction Kit (Axygen Biosciences, Union City, CA, United States). Products were checked by 2% agarose gel electrophoresis and quantified using a Quantus™ Fluorometer (Promega, United States).

Purified PCR products were used to construct sequencing libraries with the NEXTFLEX Rapid DNA-Seq Kit, following the manufacturer’s protocol: (1) adapter ligation, (2) magnetic bead selection to remove self-ligated fragments, (3) PCR enrichment of library templates, and (4) bead-based purification of the final library. Sequencing was carried out on the Illumina MiSeq PE300 platform (Shanghai Majorbio Bio-Pharm Technology Co., Ltd., China). Raw sequence data have been deposited in the China National GeneBank DataBase (CNGBdb) under accession number CNP0008191.

### 2.3 Bioinformatics analysis

Raw paired-end reads were quality-filtered using fastp version 0.19.6^1^ (fastp -i raw_R1.fq -I raw_R2.fq -o clean_R1.fq -O clean_R2.fq –cut_right –cut_window_size 50 –cut_mean_quality 20 –length_required 50 –n_base_limit 0 –disable_adapter_trimming –disable_trim_poly_g -w 4 -j fastp.json -h fastp.html) (Chen et al., 2018) and merged with FLASH version 1.2.11^2^ (flash clean_R1.fq clean_R2.fq -o merged -m 10 -M 100 -x 0.2 -t 4) (Magoč and Salzberg, 2011). The filtering steps included: (1) trimming bases with quality scores below 20 from the 3’ end of reads using a 50 bp sliding window; discarding reads shorter than 50 bp after trimming or containing ambiguous bases (N); (2) merging paired-end reads based on overlap, with a minimum overlap length of 10 bp; (3) allowing a maximum mismatch rate of 0.2 in the overlap region, discarding sequences exceeding this threshold; and (4) assigning sequences to samples according to unique barcodes and primer sequences (no mismatches allowed for barcodes, up to two mismatches permitted for primers).

Quality-filtered sequences were processed using the UPARSE pipeline (version 7.1) (Edgar, 2013) as follows: (1) Dereplication of quality-filtered sequences was performed using the -fastx_uniques command with the -sizeout option to retain abundance information; (2) OTU clustering at 97% similarity was conducted using the -cluster_otus command, with the -minsize 2 option to remove singletons, which simultaneously performed chimera removal; (3) An OTU table was generated by mapping quality-filtered reads to the OTU sequences using the -otutab command with -id 0.97; (4) Taxonomic assignment of OTUs was performed using the -sintax command against the RDP 16S rRNA gene database (version 16) with a cutoff of 0.8; (5) OTUs classified as chloroplasts or mitochondria were excluded from downstream analyses. To ensure comparability across samples, the OTU table was rarefied to the minimum sequencing depth (*n* = 38,642 sequences per sample) using the rrarefy function in the vegan package (version 2.6–4) in R. This rarefaction depth yielded an average Good’s coverage of 99.89%, indicating sufficient sequencing depth for reliable diversity analyses.

Taxonomic classification of representative OTU sequences was performed using the RDP Classifier version 2.11^3^ (Wang et al., 2007) against the Silva 16S rRNA database version 138, with a confidence threshold of 70% (parameter: -c 0.7). Community composition was summarized at different taxonomic levels (phylum, class, order, family, genus) based on the classification results.

All diversity analyses were performed on the Majorbio Cloud Platform.^4^ Alpha diversity indices, including Chao and Shannon, were calculated using mothur^5^ (Schloss et al., 2009). Differences in alpha diversity between groups were assessed with the Wilcoxon rank-sum test. Beta diversity was evaluated by principal coordinates analysis (PCoA) based on Bray–Curtis distances. Group-level differences in microbial community structure were further tested using PERMANOVA (permutational multivariate analysis of variance).

Differentially abundant taxa from phylum to genus levels were identified with LEfSe (linear discriminant analysis effect size)^6^ (Segata et al., 2011) using LDA > 4.0 and *P* < 0.05 as significance thresholds. Distance-based redundancy analysis (db-RDA) was performed to explore the influence of environmental factors on gut microbial community composition.

## 3 Results

### 3.1 Sequencing quality and OTU statistics

Through Illumina MiSeq sequencing, we obtained a total of 1,368,262 high-quality 16S rRNA sequence reads from the 30 gut samples (averaging 45,609 reads per sample). After quality filtering and processing, the average read length was 428 bp. Good’s coverage values for all samples exceeded 0.99, indicating that the sequencing depth was sufficient to capture the majority of the bacterial diversity in the gut communities.

Clustering at 97% sequence similarity yielded 388 representative OTUs, of which 368 remained after removing low-abundance and potentially biased OTUs. These 368 OTUs were distributed across 25 phyla, 51 classes, 104 orders, 163 families, 225 genera, and 331 species, based on phylogenetic classification of the 30 gut samples. The number of OTUs identified per sample is summarized in Table 1. The sequencing accuracy was high, ensuring that the data met the standards for downstream analyses.

**TABLE 1:** Number of bacterial taxa in the gut of *C. kiangsu* from different geographical populations.

Sample	OTU	Species	Genus	Family	Order	Class	Phylum
CB01	30	30	27	22	14	4	3
CB02	23	23	23	18	15	5	4
CB03	29	29	27	22	14	5	3
CB04	28	28	23	18	16	7	5
CB05	18	17	16	14	12	8	4
CZ01	27	27	23	22	15	7	3
CZ02	26	26	24	21	14	6	4
CZ03	58	55	49	46	39	26	15
CZ04	28	28	26	23	17	10	6
CZ05	25	25	23	20	14	6	4
GD01	93	89	71	48	28	10	6
GD02	39	37	31	23	15	6	5
GD03	38	37	32	28	19	9	6
GD04	57	56	44	33	19	7	6
GD05	124	120	99	67	44	18	13
GX01	42	42	35	27	17	6	4
GX02	19	17	16	11	7	4	3
GX03	42	41	36	28	18	6	4
GX04	37	36	29	24	20	10	7
GX05	17	15	13	9	4	3	3
YN01	21	17	15	13	9	4	3
YN02	14	14	12	10	8	5	4
YN03	25	22	19	16	13	5	4
YN04	14	12	11	9	7	4	3
YN05	32	30	25	21	14	5	4
YY01	42	42	36	31	24	12	7
YY02	6	6	6	5	5	4	3
YY03	4	4	4	3	3	2	2
YY04	42	41	33	27	16	9	6
YY05	47	46	43	37	25	13	8

### 3.2 Gut microbiota diversity and composition among populations

**FIGURE 2:**
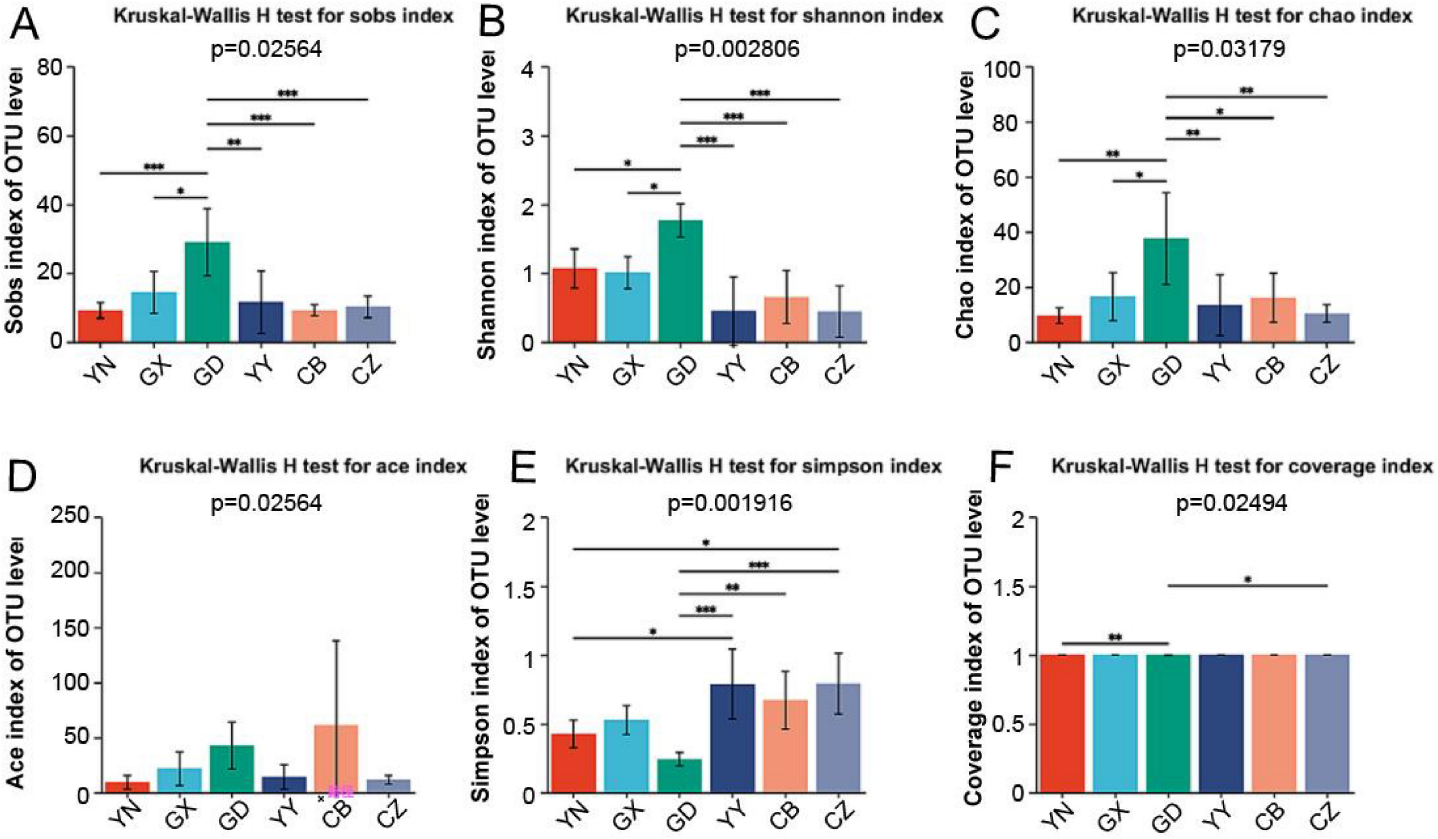
Alpha diversity indices of gut microbial communities in *C. kiangsu* from different geographical populations. **(A)** Sobs index, **(B)** Shannon index, **(C)** Chao index, **(D)** Ace index, **(E)** Simpson index, and **(F)** Coverage index. *P < 0.05, **P < 0.01, ***P < 0.001.

Microbial diversity varied considerably among the six geographical *C. kiangsu* populations. The observed OTU counts and alpha-diversity indices differed by region. In general, the GD population exhibited the highest gut microbiota diversity, whereas some other populations showed lower diversity. For instance, GD had the greatest number of observed OTUs and the highest Chao1 richness and Shannon diversity indices, significantly higher than those of several other populations (Figure 2 and Supplementary Table 1). In contrast, the Yiyang, Hunan (YY) population had among the lowest diversity indices. Notably, the Simpson index (which measures community evenness) was lowest in GD, indicating that although GD harbored more diverse bacteria, a few taxa dominated its community. These results suggest that the gut microbiome of the GD population is more complex, while certain other populations host less diverse communities.

**FIGURE 3:**
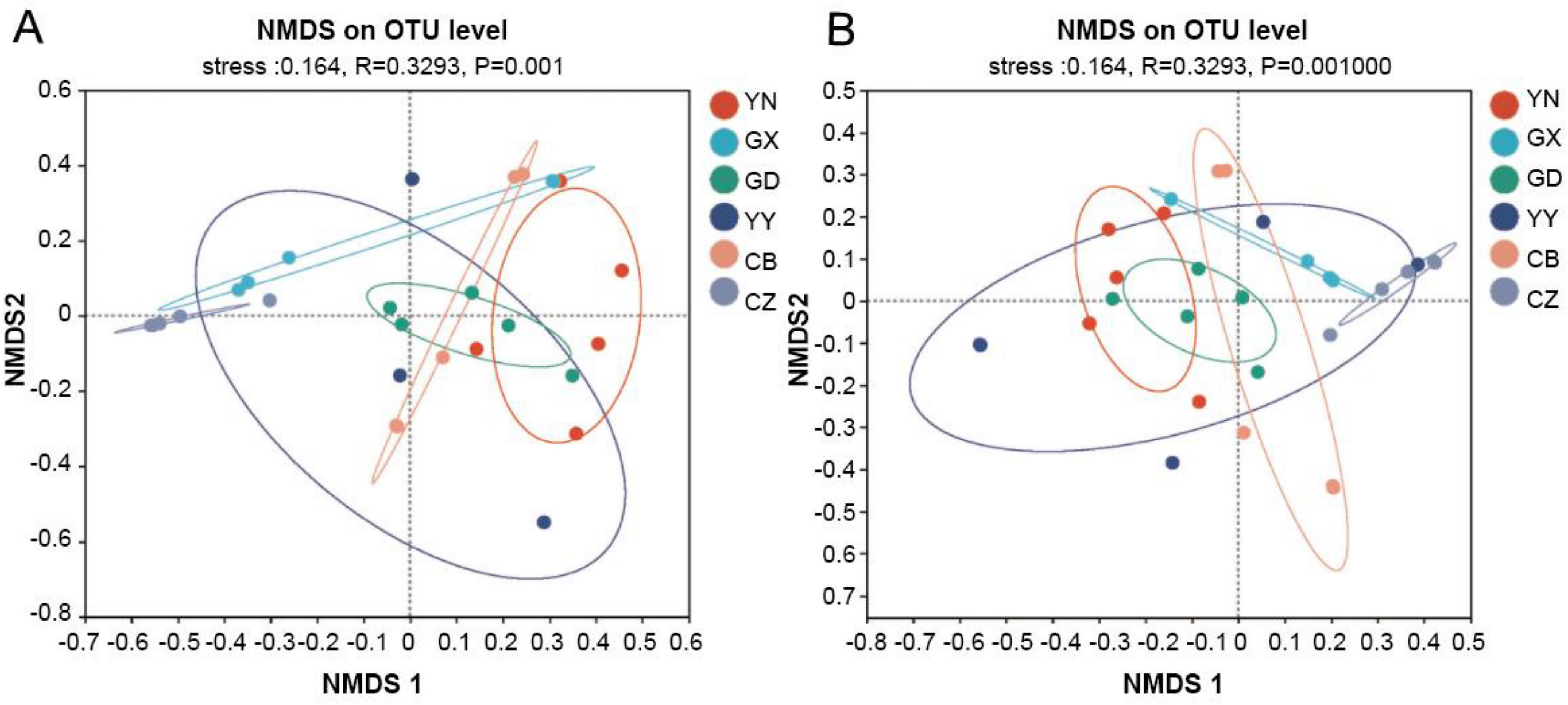
Gut microbial diversity of *C. kiangsu* from different geographical populations. Principal coordinate analysis (PCoA) and non-metric multidimensional scaling (MDS) based on Bray–Curtis distance of bacterial abundance are shown in **(A,B)**, respectively.

Beta diversity analysis further demonstrated that each geographic population harbored a distinct gut microbial community structure. Principal coordinates analysis (PCoA) of Bray–Curtis distances revealed clear separation of samples according to their population of origin (Figure 3A). Similarly, non-metric multidimensional scaling (NMDS) showed that samples clustered by location (Figure 3B). These patterns indicate that the composition of the gut microbiota differs markedly from one population to another. An analysis of similarity confirmed that the between-population differences were statistically significant (ANOSIM: *R* = 0.3293, *p* = 0.001). In summary, both alpha- and beta-diversity results consistently suggest that gut microbial communities of *C. kiangsu* differ significantly among geographical populations.

**FIGURE 4:**
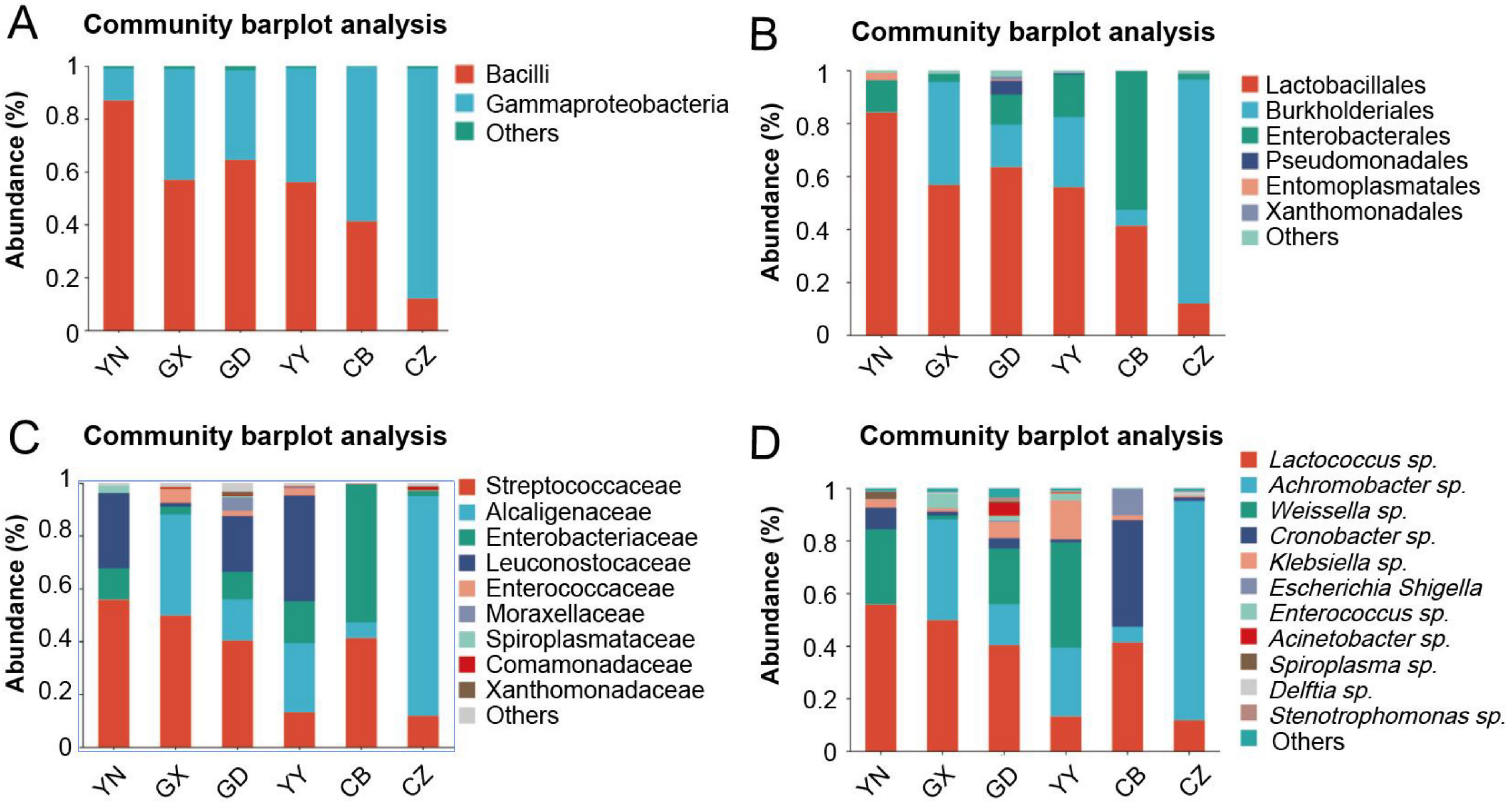
Relative abundance of gut bacterial communities in *C. kiangsu* from different geographical populations at the class **(A)**, order **(B)**, family **(C)**, and genus **(D)** levels. Each color represents one taxon in the corresponding chart. “Others” indicates taxa not listed at the respective taxonomic level.

Taxonomic composition analysis illustrated the specific differences in community makeup across the populations. Overall, the gut microbiota was dominated by two phyla, Firmicutes and Proteobacteria, in all samples (Supplementary Figure 1). However, the relative balance between these dominant phyla varied by region. For example, Firmicutes reached the highest relative abundance in the YN population (accounting for up to ∼87% of sequences), whereas Proteobacteria predominated in the Hubei (CB) population (up to ∼87%). Other phyla, such as Bacteroidota and Actinobacteriota, were present at much lower abundances. At the genus level, the most abundant taxa also differed among populations. *Lactococcus* (a lactic acid bacterium in the family Streptococcaceae) was particularly dominant in YN, comprising over half of the gut bacteria in that population, while *Achromobacter* (a genus of *Alcaligenaceae* in Proteobacteria) was highly prevalent in the CZ population. Other genera among the top ten in abundance included *Weissella*, *Cronobacter*, *Klebsiella*, *Enterococcus*, *Escherichia–Shigella*, *Spiroplasma*, and *Delftia*, each showing distinct distribution patterns across the six populations (Figure 4). These compositional differences align with the diversity analyses above, reinforcing that each geographic population of *C. kiangsu* harbors a unique gut microbiota profile.

### 3.3 LDA analysis of gut microbiota in *C. kiangsu* from different geographical populations

**FIGURE 5:**
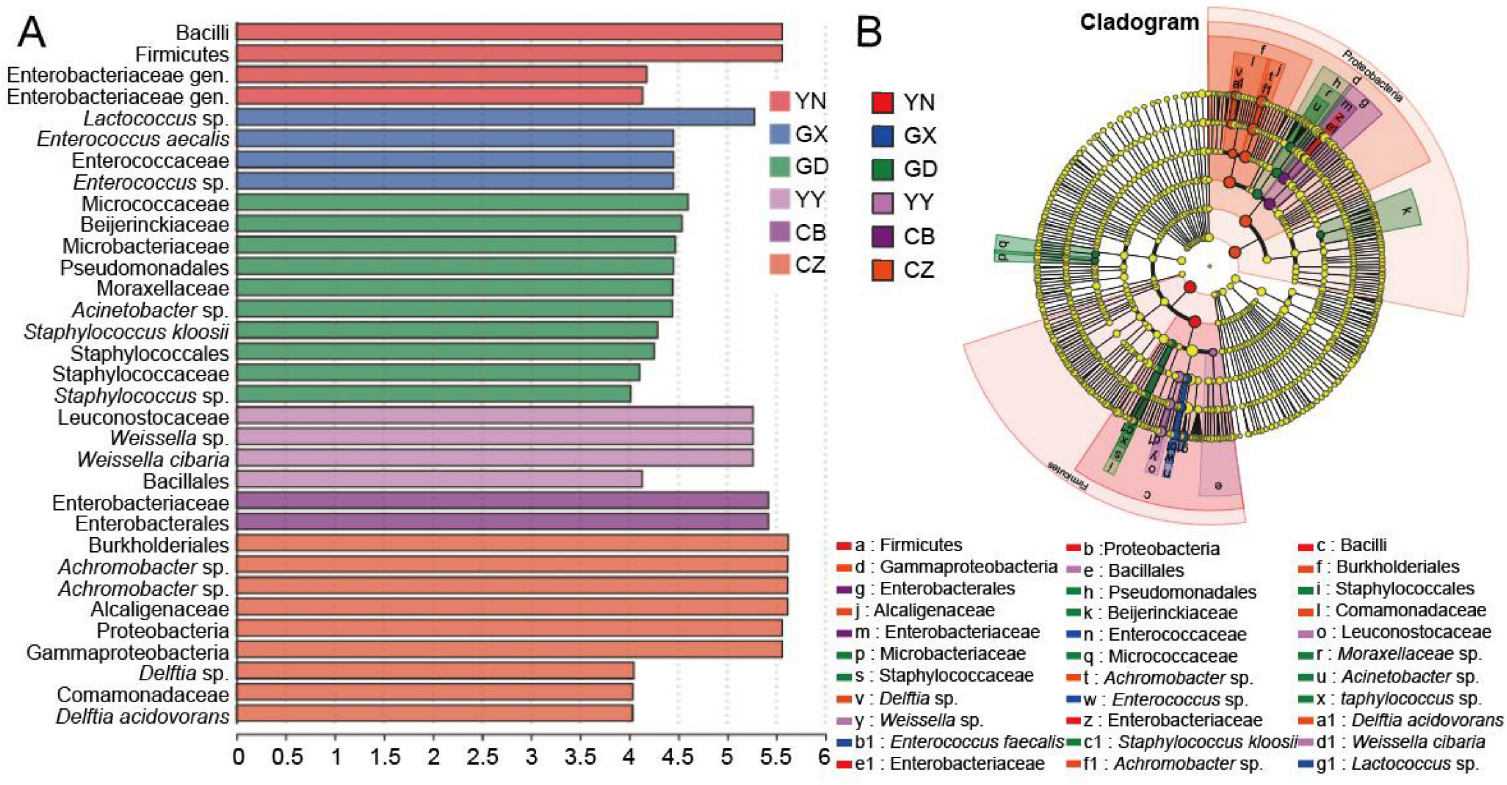
LDA analysis of gut microbiota in *C. kiangsu* from different geographical. **(A)** Bacterial taxa with LDA scores > 4 in the gut microbiota of *C. kiangsu* from different geographical populations. **(B)** Evolutionary cladogram of bacterial biomarkers from phylum (innermost ring) to species (outermost ring) levels with LDA scores > 4. Different bacterial taxa are marked with lowercase letters. Each small circle represents one taxon at the corresponding taxonomic level, and the diameter of the circle is proportional to its relative abundance. Nodes with different colors indicate taxa that are significantly enriched in the corresponding groups and have a significant impact on intergroup differences, while light yellow nodes represent taxa with no significant differences among groups or with no significant contribution to intergroup variation.

We identified a total of 33 discriminative bacterial taxa whose abundances differed significantly among the six populations using LEfSe (linear discriminant analysis effect size) with LDA > 4.0 (Figure 5). The majority of these differentially abundant taxa belonged to the phyla Proteobacteria, Firmicutes, or Actinobacteriota. The GD population had the largest set of enriched taxa (10 in total), including four taxa affiliated with Proteobacteria, four with Firmicutes, and two with Actinobacteriota. Notably, GD was the only population in which Actinobacteriota (specifically two genera from that phylum) were detected as significant biomarkers. The YN population showed four biomarker taxa (two Proteobacteria and two Actinobacteriota). In the GX population, two genera of Proteobacteria were identified as significantly enriched. The YY population had four enriched taxa, all belonging to Proteobacteria. The CB population exhibited two Proteobacteria biomarkers. Finally, the Chenzhou, Hunan (CZ) population had nine distinct indicator taxa, and all of them were classified within Proteobacteria. These LEfSe results highlight which specific bacteria differentiate each population’s gut microbiota.

### 3.4 Correlation between ecological factors and gut microbiota structure of *C. kiangsu*

We next analyzed how environmental differences among the collection sites might relate to the observed gut microbiota variation. Meteorological data indicated substantial differences in climate variables such as temperature, rainfall, and sunlight among the six locations (Table 2). Spearman correlation analyses suggested that several alpha-diversity indices had significant relationships with certain environmental factors (Supplementary Figure 2). For example, the Shannon and Simpson diversity indices of the gut microbiota were significantly correlated with mean temperature (T), precipitation (P), humidity (H), evaporation (E), and ground temperature (Gt). The Chao1 richness index showed a significant positive correlation with atmospheric pressure (Pa), whereas the Good’s coverage index was correlated with sunshine duration (S) and Pa.

**TABLE 2:** Statistical data of average meteorological conditions in different geographical locations in 2022.

Sample	Temperature 2 m (°C)	Precipitation (mm)	Relative humidity (%)	Sunshine duration (Peak, h)	Wind speed 10 m (m/s)	Evaporation (mm)	Surface air pressure (hPa)	Surface temperature (°C)
	**T**	**P**	**H**	**S**	**W**	**E**	**Pa**	**GT**
**GX**	17.48	2083.31	74.86	1261.56	2.21	764.77	947.76	17.21
**GD**	21.14	2255.33	76.59	1376.4	2.04	1053.63	983.26	20.5
**YY**	17.35	1618.0	72.17	1370.96	2.43	890.17	977.13	17.1
**CZ**	18.26	1656.91	74.14	1323.0	1.81	707.39	953.54	18.09
**YN**	20.94	1877.71	79.47	1625.68	1.36	1034.7	890.73	19.5
**CB**	18.01	1279.61	70.51	1414.54	2.21	881.65	993.69	17.73

Temperature (T), precipitation (P), sunshine (S), and wind (W) at the six sampling sites of *C. kiangsu*: YN, Mengla (Yunnan); GD, Guangning (Guangdong); GX, Xing’an (Guangxi); CZ, Suxian (Chenzhou, Hunan); YY, Taojiang (Yiyang, Hunan); CB, Chibi (Hubei).

To reduce multicollinearity among these interrelated variables, we performed a variance inflation factor (VIF) analysis and excluded redundant factors (VIF > 10) (Zhang et al., 2023) before further analysis. As a result, temperature (T), precipitation (P), sunshine duration (S), and wind (W) were retained for further analysis. Using canonical correspondence analysis (CCA) with the remaining variables, we found that precipitation and sunshine duration were the environmental factors most strongly associated with differences in gut microbiota composition across the populations. In the CCA, annual precipitation and sunlight explained a significant proportion of the variation in community structure (permutation test: *P* = 0.004 for precipitation, *P* = 0.017 for sunshine), with precipitation showing the highest explanatory power (CCA R^2^ ≈ 0.347) (Supplementary Figure 3 and Table 3). These results indicate that sites with different rainfall and sunlight regimes tend to host correspondingly different gut microbial communities in *C. kiangsu*.

**TABLE 3:** Parameters of canonical correspondence analysis (CCA).

Environmental factors	CCA1	CCA2	r2	*P*-value
Temperature (T)	−0.93	0.3675	0.1358	0.137
Precipitation (P)	−0.0343	0.9994	0.3471	0.004
Sunshine (S)	−0.9918	−0.1277	0.2743	0.017
Wind (W)	0.3691	−0.9294	0.0127	0.843

**FIGURE 6:**
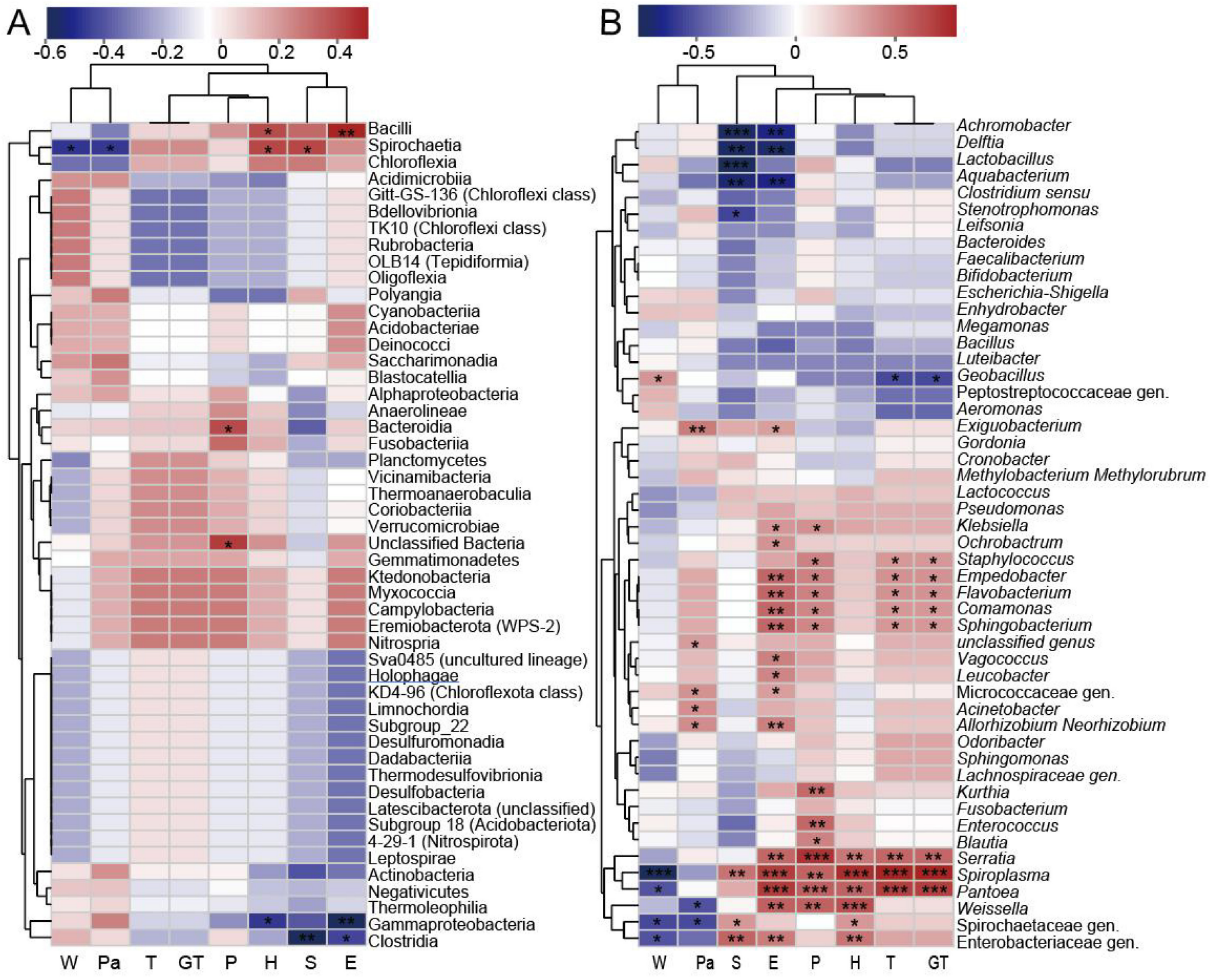
Geographic factor analysis of gut microbiota in different populations of *C. kiangsu* at the class **(A)** and genus **(B)** level. *P < 0.05, **P < 0.01, ***P < 0.001.

We also explored correlations between specific bacterial taxa and the key environmental factors. At the phylum level (Supplementary Figure 1), considering that the relative abundances of Firmicutes and Proteobacteria are extremely high, while the abundances of other phyla are very low, correlation analysis between minor taxa and environmental factors may yield statistically unreliable or unexplainable results. Therefore, no correlation analysis with environmental factors will be performed at the phylum level. At the class level (Figure 6A), W and S were correlated with Spirochaetia, P with Bacteroidia and unclassified classes, whereas T showed no significant associations.

At the order level (Supplementary Figure 4), W correlated with Entomoplasmatales and Spirochaetales, while S was associated with multiple orders, including Peptostreptococcales-Tissierellales, Burkholderiales, Lachnospirales, Bacillales, Xanthomonadales, Enterobacterales, and Spirochaetales. T was linked to Entomoplasmatales, Sphingomonadales, Pseudomonadales, Flavobacteriales, Sphingobacteriales, Staphylococcales, and Competibacterales. P showed significant associations with Entomoplasmatales, Caulobacterales, Pseudomonadales, Flavobacteriales, Sphingobacteriales, Staphylococcales, Competi- bacterales, and unclassified orders.

At the family level (Supplementary Figure 5), W was correlated with Spiroplasmataceae, Erwiniaceae, and Spirochaetaceae. S was associated with Spiroplasmataceae, Spirochaetaceae, Enterobacteriaceae, Alcaligenaceae, Comamona- daceae, Lactobacillaceae, Cyclobacteriaceae, Lachnospiraceae, and Xanthomonadaceae. T was linked with Spiroplasmataceae, Yersiniaceae, Erwiniaceae, Beijerinckiaceae, Sphingomonadaceae, Weeksellaceae, Staphylococcaceae, Flavobacteriaceae, and Sphingobacteriaceae. P was associated with Spiroplasmataceae, Yersiniaceae, Erwiniaceae, Enterococcaceae, Planococcaceae, Leuconostocaceae, Weeksellaceae, Staphylococcaceae, Flavo- bacteriaceae, and Sphingobacteriaceae.

At the genus level (Figure 6B), W correlated with *Spiroplasma*, *Pantoea*, *Geobacillus*, and unclassified genera within *Spirochaetaceae* and *Enterobacteriaceae*. S was associated with *Spiroplasma*, *Stenotrophomonas*, and unclassified genera of *Spirochaetaceae* and *Enterobacteriaceae*. T was significantly related to *Spiroplasma*, *Pantoea*, *Geobacillus*, *Serratia*, *Staphylococcus*, *Empedobacter*, *Flavobacterium*, *Comamonas*, and *Sphingobacterium*. P showed significant correlations with *Spiroplasma*, *Pantoea*, *Kurthia*, *Enterococcus*, *Blautia*, *Serratia*, *Weissella*, and *Klebsiella*.

## 4 Discussion

This study provides a comprehensive analysis of the gut microbial communities of *C. kiangsu* across six geographically distinct populations in China. We found clear differences in both the diversity and taxonomic composition of gut bacteria among these populations, and these differences were significantly associated with regional environmental factors—particularly precipitation and sunshine duration. These findings reinforce the general ecological principle that insect gut microbiota are shaped by a combination of host and environmental influences (Zhang et al., 2023; Liu et al., 2019).

At the community level, we confirmed that Firmicutes and Proteobacteria are the dominant bacterial phyla in the gut of *C. kiangsu*, which is consistent with reports in other locust and grasshopper species (Lavy et al., 2018; Wang et al., 2020). These two phyla often constitute the core microbiota in orthopteran insects, reflecting a common role in digestion and metabolism for the host. However, we also observed pronounced differences at finer taxonomic levels among populations. For instance, the gut microbiota of the YN population was overwhelmingly dominated by Lactococcus (Family Streptococcaceae, Phylum Firmicutes), suggesting this bacterium may play an important role in that population—potentially in fermentative digestion of bamboo or in pathogen defense, as lactic acid bacteria are known to produce antimicrobial compounds (Anumudu et al., 2024). In contrast, the HB population had a very high abundance of Achromobacter (Family Alcaligenaceae, Phylum Proteobacteria), which might indicate that this genus confers some adaptive advantage under local conditions—perhaps related to detoxification of plant allelochemicals or tolerance to the specific climate of that region (Jing et al., 2020). Such geographically specific microbial patterns likely represent microbiome remodeling strategies that enable hosts to cope with distinct environmental pressures (Bai et al., 2022). In other words, each *C. kiangsu* population may enrich for certain symbiotic bacteria that best help the locust utilize local food resources or withstand local stresses.

Both our alpha- and beta-diversity analyses indicated that geographic factors significantly influence gut microbial community structure in *C. kiangsu*. Among the populations we studied, the GD population (from a region with a warm, humid subtropical climate) exhibited the highest microbial richness and diversity. This suggests that a more heterogeneous or resource-rich environment may promote the development of a more complex gut microbiome. A similar observation was reported in other insects; for example, in the small brown planthopper, populations in more diverse habitats harbored more diverse gut microbiota, which was proposed to help the insect adapt to varied environments (Duan et al., 2020). Additionally, prior studies on the migratory locust noted that population differences in environmental conditions (e.g., temperature regimes) could drive differences in physiological traits (Jing and Kang, 2003), which aligns with our finding that environmental heterogeneity (as seen in GD) might correspond to greater microbial diversity. In contrast, populations from more temperate or less diverse environments (such as the Hunan populations in our study) showed lower gut microbiota diversity, which might reflect a more specialized microbiome tuned to a narrower range of conditions or diet.

Crucially, our ecological correlation analyses identified precipitation (rainfall) and sunshine duration as key environmental drivers of gut microbiota variation among the populations. Precipitation had the strongest association with changes in the gut community structure, with higher annual rainfall correlating with significant shifts in microbial composition. This finding is particularly interesting in light of historical observations that link rainfall patterns to locust outbreaks (Tian et al., 2011). It suggests that climatic factors like rainfall might influence locust populations not only directly (e.g., via habitat and food availability) but also indirectly through modulating the insect’s symbiotic gut microbiota. For instance, wetter conditions could affect the microbial communities on the host plants (bamboo) or alter the physiological state of the locusts, thereby leading to changes in the gut microbiome. Sunshine duration, a proxy for light intensity and perhaps linked to temperature, was another significant factor: environments with more sunlight were associated with distinct gut microbial profiles. Longer sunshine hours might correspond to higher daytime temperatures or greater ultraviolet exposure, which could influence microbial survival and growth either inside the host or in the external environment from which the gut microbes are acquired.

We found that certain gut bacterial taxa were more abundant under specific environmental conditions (for example, *Spiroplasma* tended to be more abundant in locusts from high-precipitation or high-sunshine areas). *Spiroplasma* is a genus of Mollicutes known to include insect endosymbionts that can influence host biology, such as by enhancing resistance to parasites or affecting reproduction (Xie et al., 2010). Its association with both precipitation and sunshine in our study raises questions about its functional role—perhaps *Spiroplasma* provides fitness benefits under climatic stress, or its transmission is easier in certain weather conditions. Similarly, *Achromobacter* and *Cronobacter*, which we observed at relatively high abundances in multiple populations despite being rarely reported in other locust species, may have functional significance. These genera are known from environmental sources and some can degrade complex compounds; thus, they might help *C. kiangsu* digest specific components of bamboo or cope with chemical defenses of the plant. Their consistent presence across geographically separated populations suggests a possible long-term coevolutionary relationship between *C. kiangsu* and its bamboo diet or habitat microbiota. For instance, *Achromobacter* has been implicated in the degradation of certain pesticides and could aid in detoxifying plant allelochemicals (Xia et al., 2017), while *Cronobacter* (though often known as an opportunistic pathogen in other contexts) might be involved in nutrient acquisition from the bamboo (Jing et al., 2020). *Spiroplasma*, as noted, could be providing defensive benefits to the locust. These hypotheses require further investigation, but they illustrate how examining the microbiome can yield insights into the ecological adaptation of the host insect.

In summary, our study reveals clear geographic patterns in the gut microbiota of *C. kiangsu* and highlights the potential roles of precipitation and sunshine duration in shaping these microbial communities. These findings provide a microbiological basis for understanding the ecological adaptability and population dynamics of *C. kiangsu*. Practically, the results suggest that microbial indicators could be considered when assessing locust population health or predicting outbreak risk under varying climatic conditions. From an applied perspective, if certain symbionts are found to confer advantages to the locust (for example, aiding nutrition or stress tolerance), disrupting those symbionts might offer a novel pest management strategy.

It is also important to note the limitations of our study. First, we sampled the gut microbiota at a single time point (in mid-summer 2022) and correlated it with annual average environmental data. As such, the correlations we observed provide a snapshot that may not capture seasonal dynamics. Second, our sample size at each site was limited to five replicates (each a pool of three insects), which may not fully represent the within-population microbiota variability. Future studies should include multi-season or multi-year sampling to confirm how temporal changes in climate factors affect the gut microbiome, whereas we have only considered the average estimates available to us from the tested locations. In addition, increasing the number of samples per site would help improve the detection of microbial community differences among populations. Experimental approaches, such as reciprocal transplant experiments or controlled diet trials, could also help establish causal links between specific environmental variables and microbial community shifts. Furthermore, our analysis was based on 16S rRNA sequencing, which identifies bacteria present but not their functional activity.

Because herbivorous insects inevitably ingest plant- and environment-associated microorganisms, amplicon-based gut profiles from field-caught specimens may contain a mixture of resident, host-associated taxa and transient taxa derived from diet or local microbial reservoirs. To minimize these transient signals, we implemented a standardized 48-h starvation step prior to dissection to facilitate gut clearance, and we gently rinsed dissected gut tissues with pre-chilled sterile 1 × PBS before DNA extraction to remove residual luminal contents and loosely associated microbes—an approach commonly used in insect gut microbiome studies to reduce carryover contamination (Wang et al., 2020; Bai et al., 2022; Tutagata et al., 2024). Nevertheless, these procedures cannot fully eliminate dietary and environmental contributions. Notably, phyllosphere microbiomes are strongly structured by environmental gradients such as precipitation, humidity, and solar radiation (Xu et al., 2022); therefore, the geographic differentiation observed in *C. kiangsu* gut communities may partially reflect site-specific environmental microbial pools across sampling locations. Conversely, several studies suggest that insect gut microbiota are not necessarily strongly determined by the host plant phyllosphere microbiome (Hannula et al., 2019; Zhang et al., 2025; Yan et al., 2025). Taken together with our standardized processing workflow, we infer that the geographic patterns detected here are more likely to primarily represent responses of endogenous gut symbionts of *C. kiangsu* to environmental gradients, although we adopt a precautionary interpretation and frame our conclusions as strong associations rather than definitive causation. Future work integrating host-plant microbiome sampling and common-garden or reciprocal feeding experiments will help disentangle resident versus transient contributions and clarify causality.

To address functional questions, multi-omics approaches will be valuable. Future studies integrating metagenomics, metatranscriptomics, and metabolomics could elucidate the metabolic capabilities of key symbionts and how they contribute to host nutrition, immunity, and behavior. For example, metagenomic analyses could reveal whether microbes in high-rainfall populations carry genes for degrading certain bamboo components or resisting gut pathogens. Metabolomic profiling could show how microbial metabolites differ in locusts from different climates. Such integrated studies will provide deeper insight into the symbiotic mechanisms underlying *C. kiangsu*’s adaptation and may point to novel targets for microbiome-based pest control. Overall, our findings lay the groundwork and highlight the need for these future investigations to fully understand the complex interplay between locusts, their gut microbes, and the environment.

## Data Availability

The datasets presented in this study can be found in online repositories. The names of the repository/repositories and accession number(s) can be found at: https://db.cngb.org/data_resources/project/CNP0008191/, CNP0008191.

## Footnotes

1
https://github.com/OpenGene/fastp

2
http://www.cbcb.umd.edu/software/flash

3
https://github.com/rdpstaff/classifier

4
https://cloud.majorbio.com

5
http://www.mothur.org/wiki/Calculators

6
http://huttenhower.sph.harvard.edu/LEfSe

## References

[B1] Anumudu C. K. Miri T. Onyeaka H. (2024). Multifunctional applications of lactic acid bacteria: Enhancing safety, quality, and nutritional value in foods and fermented beverages. *Foods* 13:3714. 10.3390/foods13233714 39682785 PMC11640447

[B2] Bai J. Ling Y. Li W. J. Wang L. Xue X. B. Gao Y. Y. et al. (2022). Analysis of intestinal microbial diversity of four species of grasshoppers and determination of cellulose digestibility. *Insects* 13:432. 10.3390/insects13050432 35621768 PMC9147371

[B3] Chen F. Z. You L. J. Yang F. Wang L. N. Guo X. Q. Gao F. et al. (2020). CNGBdb: China national genebank database. *Yi Chuan* 42 799–809. 10.16288/j.yczz.20-080 32952115

[B4] Chen S. Zhou Y. Chen Y. Gu J. (2018). fastp: An ultra-fast all-in-one FASTQ preprocessor. *Bioinformatics* 34 i884–i890. 10.1093/bioinformatics/bty560 30423086 PMC6129281

[B5] Dillon M. E. Lozier J. D. (2019). Adaptation to the abiotic environment in insects: The influence of variability on ecophysiology and evolutionary genomics. *Curr. Opin. Insect. Sci.* 36 131–139. 10.1016/j.cois.2019.09.003 31698151

[B6] Duan X. Z. Sun J. T. Wang L. T. Shu X. H. Guo Y. Keiichiro M. et al. (2020). Recent infection by Wolbachia alters microbial communities in wild *Laodelphax striatellus* populations. *Microbiome* 8:104. 10.1186/s40168-020-00878-x 32616041 PMC7333401

[B7] Edgar R. C. (2013). UPARSE: Highly accurate OTU sequences from microbial amplicon reads. *Nat. Methods* 10 996–998. 10.1038/nmeth.2604 23955772

[B8] Fan Z. Jiang G. F. Liu Y. X. (2014). Population explosion in the yellow-spined bamboo locust *Ceracris kiangsu* and inferences for the impact of human activity. *PLoS One* 9:e89873. 10.1371/journal.pone.0089873 24603526 PMC3946154

[B9] Guo X. Chen F. Gao F. Li L. Liu K. You L. et al. (2020). CNSA: A data repository for archiving omics data. *Database* 2020:baaa055. 10.1093/database/baaa055 32705130 PMC7377928

[B10] Hannula S. E. Zhu F. Heinen R. Bezemer T. M. (2019). Foliar-feeding insects acquire microbiomes from the soil rather than the host plant. *Nat. Commun.* 10:1254. 10.1038/s41467-019-09284-w 30890706 PMC6425034

[B11] Huang K. Wang J. Huang J. Zhang S. Vogler A. P. Liu Q. et al. (2021). Host phylogeny and diet shape gut microbial communities within bamboo-feeding insects. *Front. Microbiol.* 12:633075. 10.3389/fmicb.2021.633075 34239504 PMC8260032

[B12] Huang S. Zhang H. (2013). The impact of environmental heterogeneity and life stage on the hindgut microbiota of *Holotrichia parallela* larvae (Coleoptera: Scarabaeidae). *PLoS One* 8:e57169. 10.1371/journal.pone.0057169 23437336 PMC3578786

[B13] Jing T. Z. Qi F. H. Wang Z. Y. (2020). Most dominant roles of insect gut bacteria: Digestion, detoxification, or essential nutrient provision? *Microbiome* 8 1–20. 10.1186/s40168-020-00823-y 32178739 PMC7077154

[B14] Jing X. H. Kang L. (2003). Geographical variation in egg cold hardiness: A study on the adaptation strategies of the migratory locust *Locusta migratoria* L. *Ecol. Entomol.* 28 151–158. 10.1046/j.1365-2311.2003.00497.x

[B15] Kang Y. Su J. Yao B. Ji W. Hegab I. M. Hanafy A. M. et al. (2020). Geometric morphometric analysis of the plateau zokor (*Eospalax baileyi*) revealed significant effects of environmental factors on skull variations. *Zoology* 140:125779. 10.1016/j.zool.2020.125779 32361214

[B16] Lavy O. Gophna U. Gefen E. Ayali A. (2018). The effect of density-dependent phase on the locust gut bacterial composition. *Front. Microbiol.* 9:3020. 10.3389/fmicb.2018.03020 30713526 PMC6345702

[B17] Li R. Jiang G. F. Dong S. Y. (2018). A head transcriptome provides insights into odorant binding proteins of the bamboo grasshopper. *Genes Genom.* 40 991–1000. 10.1007/s13258-018-0706-0 30155713

[B18] Li Y. Du T. Yao J. Chen Y. Shi L. Ze S. (2025). Transboundary dispersal dynamics of ceracris kiangsu: From source regions to migration corridors. *Insects* 16:400. 10.3390/insects16040400 40332884 PMC12027984

[B19] Liu D. Zhao S. Yang X. Wang R. Cang X. Zhang H. et al. (2021). Radar monitoring unveils migration dynamics of the yellow-spined bamboo locust (Orthoptera: Arcypteridae). *Comp. Electron. Agriculture* 187:106306. 10.1016/j.compag.2021.106306

[B20] Liu W. Zhang X. Wu N. Ren Y. Wang X. (2019). High diversity and functional complementation of alimentary canal microbiota ensure small brown planthopper to adapt different biogeographic environments. *Front. Microbiol.* 10:2953. 10.3389/fmicb.2019.02953 32010074 PMC6978774

[B21] Magoč T. Salzberg S. L. (2011). FLASH: Fast length adjustment of short reads to improve genome assemblies. *Bioinformatics* 27 2957–2963. 10.1093/bioinformatics/btr507 21903629 PMC3198573

[B22] Schloss P. D. Westcott S. L. Ryabin T. Hall J. R. Hartmann M. Hollister E. B. et al. (2009). Introducing mothur: Open-source, platform-independent, community-supported software for describing and comparing microbial communities. *Appl. Environ. Microbiol.* 75 7537–7541. 10.1128/AEM.01541-09 19801464 PMC2786419

[B23] Segata N. Izard J. Waldron L. Gevers D. Miropolsky L. Garrett W. S. et al. (2011). Metagenomic biomarker discovery and explanation. *Genome Biol.* 12:R60. 10.1186/gb-2011-12-6-r60 21702898 PMC3218848

[B24] Tanaka S. Zhu D. H. (2008). Geographic variation in embryonic diapause, cold-hardiness and life cycles in the migratory locust *Locusta migratoria* (Orthoptera: Acrididae) in China. *Entomol. Sci.* 11 327–339. 10.1111/j.1479-8298.2008.00284.x

[B25] Tian H. Stige L. C. Cazelles B. Kausrud K. L. Svarverud R. Stenseth N. C. et al. (2011). Reconstruction of a 1,910-y-long locust series reveals consistent associations with climate fluctuations in China. *Proc. Natl. Acad. Sci. U. S. A.* 108 14521–14526. 10.1073/pnas.1100189108 21876131 PMC3167559

[B26] Tutagata J. Pocquet N. Trouche B. Reveillaud J. (2024). Dissection of mosquito ovaries, midgut, and salivary glands for microbiome analyses at the organ level. *J. Vis. Exp.* e67128. 10.3791/67128 39431768

[B27] Wang J. M. Bai J. Zheng F. Y. Ling Y. Li X. Wang J. et al. (2020). Diversity of the gut microbiome in three grasshopper species using 16S rRNA and determination of cellulose digestibility. *PeerJ* 8:e10194. 10.7717/peerj.10194 33194406 PMC7649011

[B28] Wang Q. Garrity G. M. Tiedje J. M. Cole J. R. (2007). Naive Bayesian classifier for rapid assignment of rRNA sequences into the new bacterial taxonomy. *Appl. Environ. Microbiol.* 73 5261–5267. 10.1128/AEM.00062-07 17586664 PMC1950982

[B29] Xia Z. Y. Zhang L. Zhao Y. Yan X. Li S. P. Gu T. et al. (2017). Biodegradation of the herbicide 2,4-Dichlorophenoxyacetic acid by a new isolated strain of *Achromobacter sp*. LZ35. *Curr. Microbiol.* 74 193–202. 10.1007/s00284-016-1173-y 27933337

[B30] Xie J. Vilchez I. Mateos M. (2010). Spiroplasma bacteria enhance survival of *Drosophila hydei* attacked by the parasitic wasp *Leptopilina heterotoma*. *PLoS One* 5:e12149. 10.1371/journal.pone.0012149 20730104 PMC2921349

[B31] Xu N. Zhao Q. Zhang Z. Zhang Q. Wang Y. Qin G. et al. (2022). Phyllosphere microorganisms: Sources, drivers, and their interactions with plant hosts. *J. Agric Food Chem.* 70 4860–4870. 10.1021/acs.jafc.2c01113 35435673

[B32] Yan H. Wang E. Wei G. S. Xu X. Hurst M. R. H. Zhang B. (2025). Microbial dynamics across tri-trophic systems: Insights from plant-herbivore-predator interactions. *FEMS Microbiol. Ecol.* 101:fiaf065. 10.1093/femsec/fiaf065 40569658 PMC12199696

[B33] Yu H. Du C. M. Shi M. R. Feng L. Fu D. Y. Xu J. et al. (2021). The diversity and function of intestinal microorganisms in four geographic *Cephalcia chuxiongica* (a pine defoliator) populations. *J. Appl. Entomol.* 145 394–405. 10.1111/jen.12858

[B34] Yu Y. Wang Y. Li H. Yu X. Shi W. Zhai J. (2021). Comparison of microbial communities in colorado potato beetles (*Leptinotarsa decemlineata* Say) collected from different sources in China. *Front. Microbiol.* 12:639913. 10.3389/fmicb.2021.639913 33815327 PMC8017321

[B35] Zeng Y. Zhu D. H. (2014). Geographical variation in body size, development time, and wing dimorphism in the cricket *Velarifictorus micado* (Orthoptera: Gryllidae). *Ann. Entomol. Soc. Am.* 107 1066–1071. 10.1603/AN14040

[B36] Zhang P. Lu W. Yue L. Zhang Z. Shao X. (2025). Guava root exudate-driven rhizosphere microorganisms changes transmitted to foliar-feeding insects influence their feeding behaviour. *Plant Biotechnol. J.* 23 2963–2977. 10.1111/pbi.70109 40333527 PMC12205883

[B37] Zhang Y. Liu S. Huang X.-Y. Zi H.-B. Gao T. Ji R.-J. et al. (2023). Altitude as a key environmental factor shaping microbial communities of tea green leafhoppers (*Matsumurasca onukii*). *Microbiol. Spectr.* 11:e0100923. 10.1128/spectrum.01009-23 37921460 PMC10714740

