# Supplementary material for: Geographical variation of Ceracris kiangsu gut microbiota and its association with environmental factors
Source: Front Microbiol. 2026 Mar 20;17:1752887. doi: 10.3389/fmicb.2026.1752887 (PMC13047058; doi:10.3389/fmicb.2026.1752887)
Supplement: Supplementary file 1 [file Data_Sheet_1.docx]

Supplementary Material

# Supplementary Figures and Tables

## Supplementary Figures


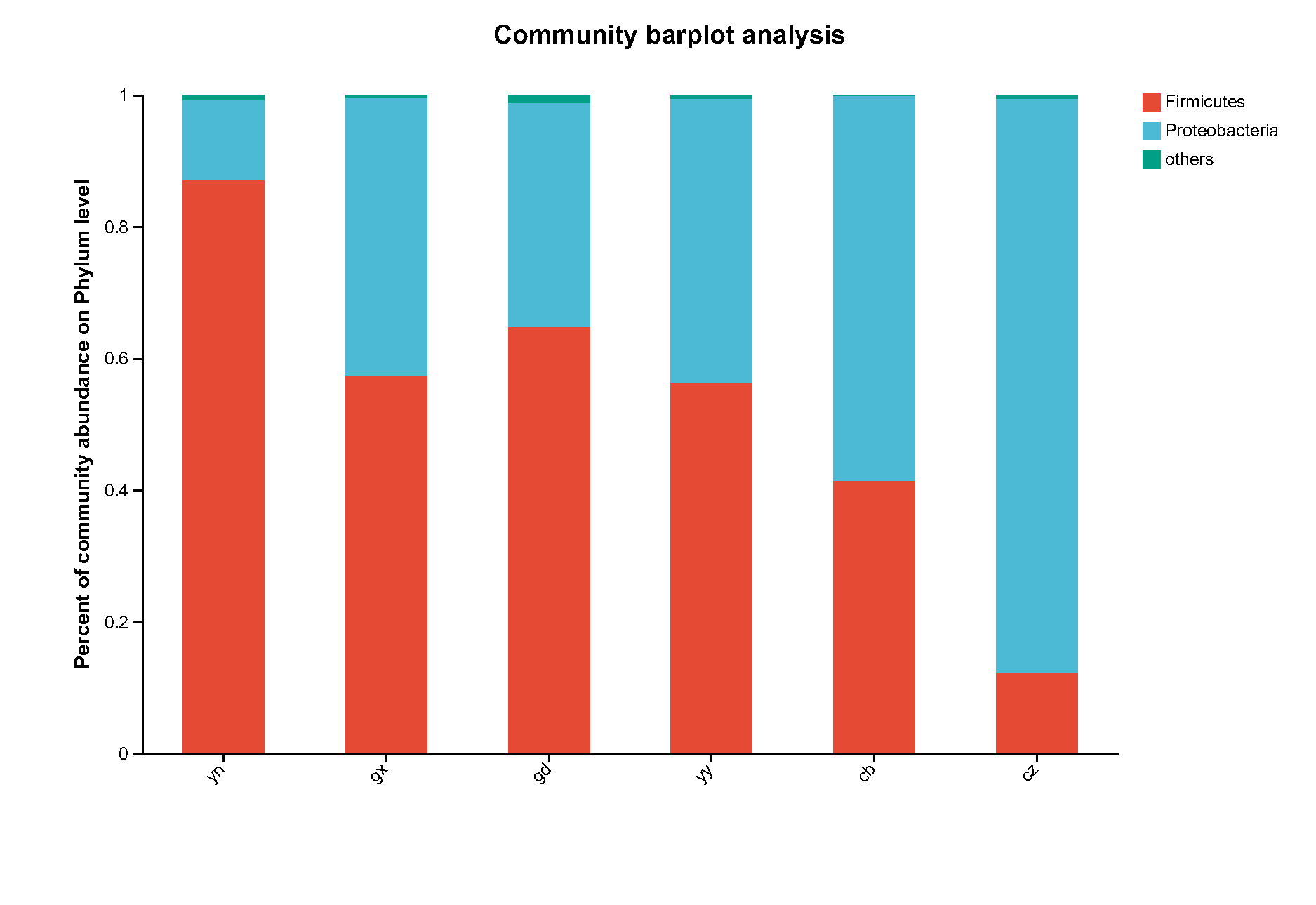


**Supplementary Figure 1.** Relative abundance of gut bacterial communities in *C. kiangsu* from different geographical populations at the phylum levels. Each color represents one taxon in the corresponding chart. “Others” indicates taxa not listed at the respective taxonomic level.


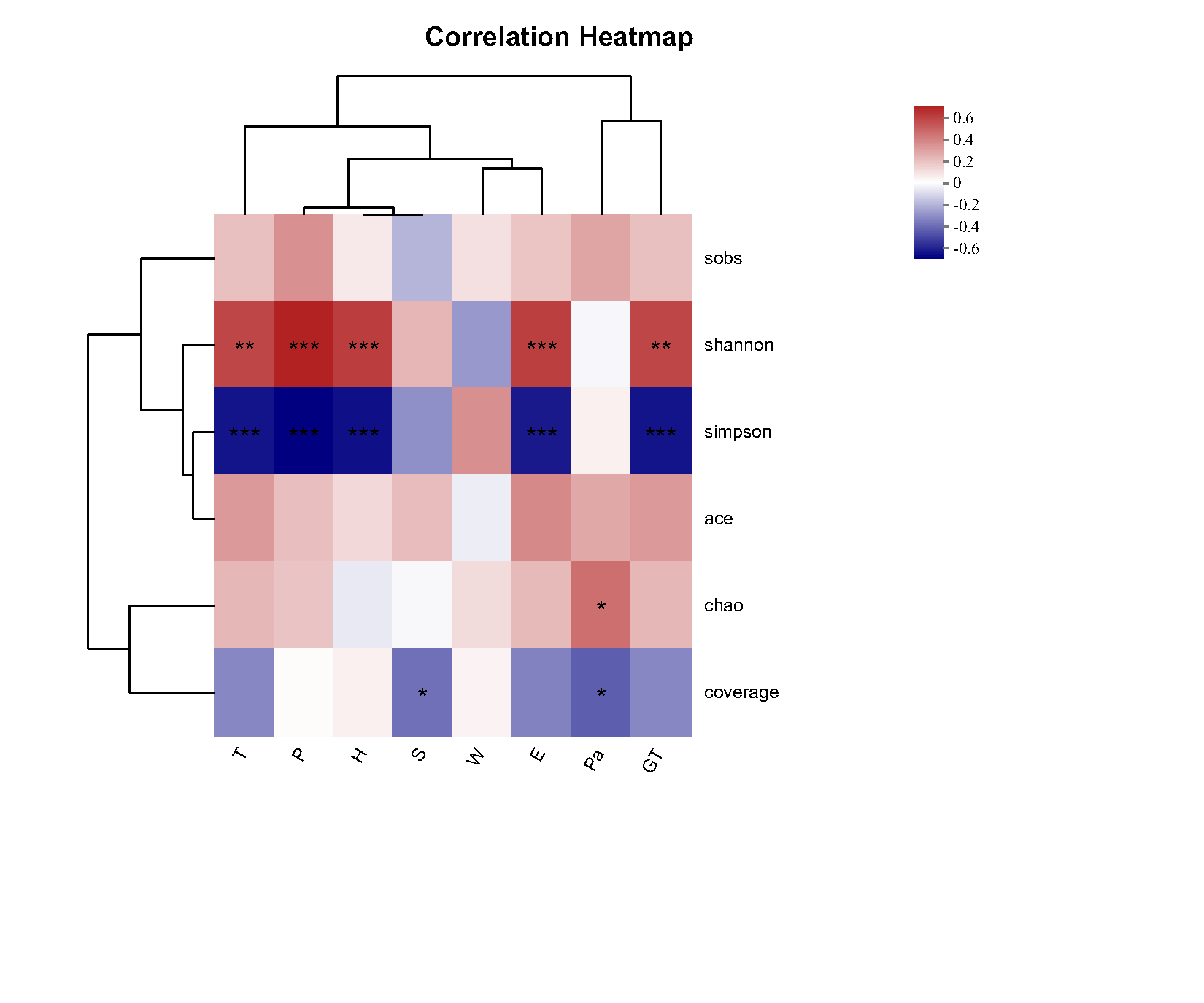


**Supplementary Figure 2.** Analysis of Environmental Factors in Different Geographical Populations of *C. kiangsu*


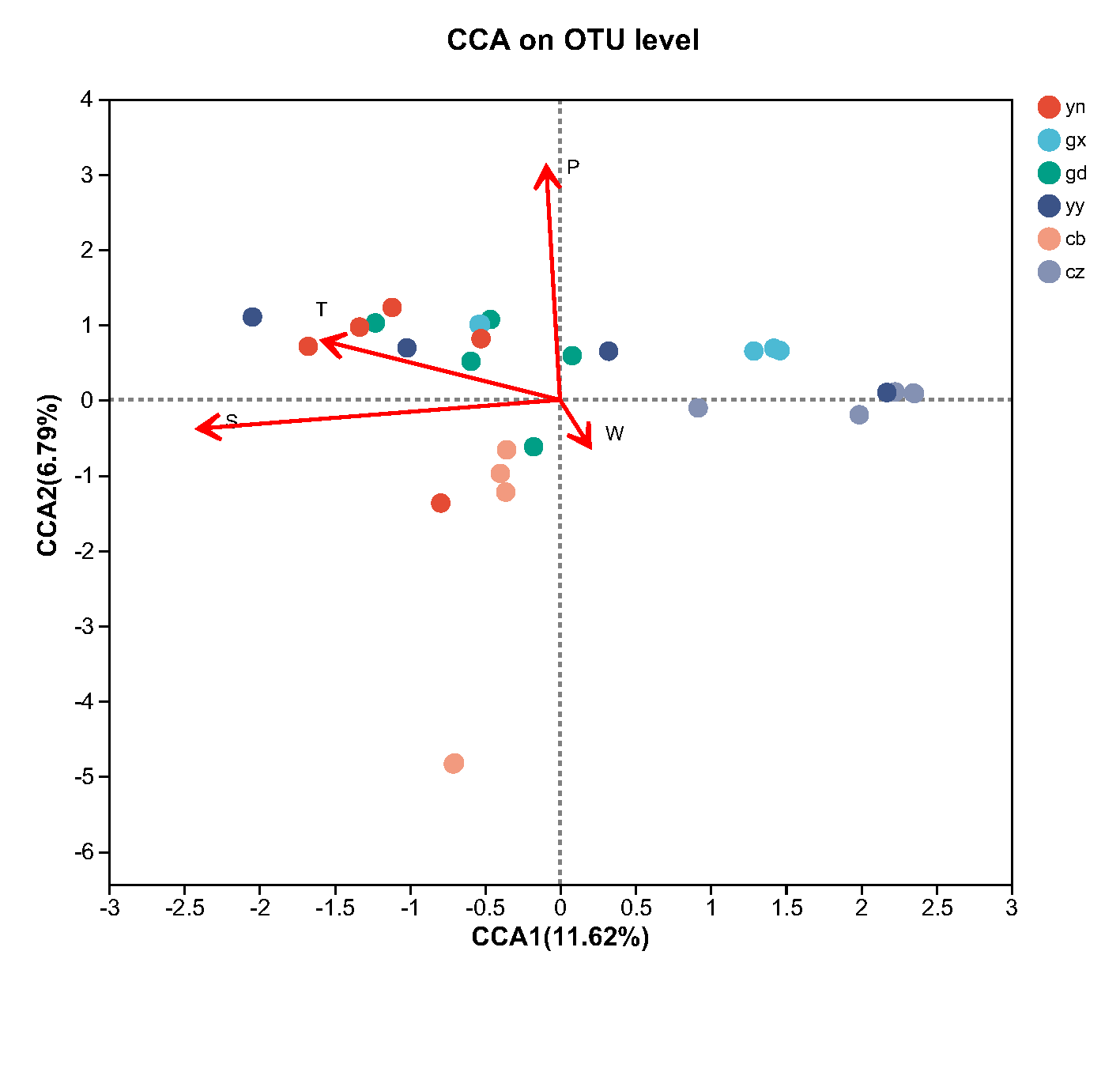


**Supplementary Figure 3.** Analysis of Environmental Factors in Different Geographical Populations of *C. kiangsu.*


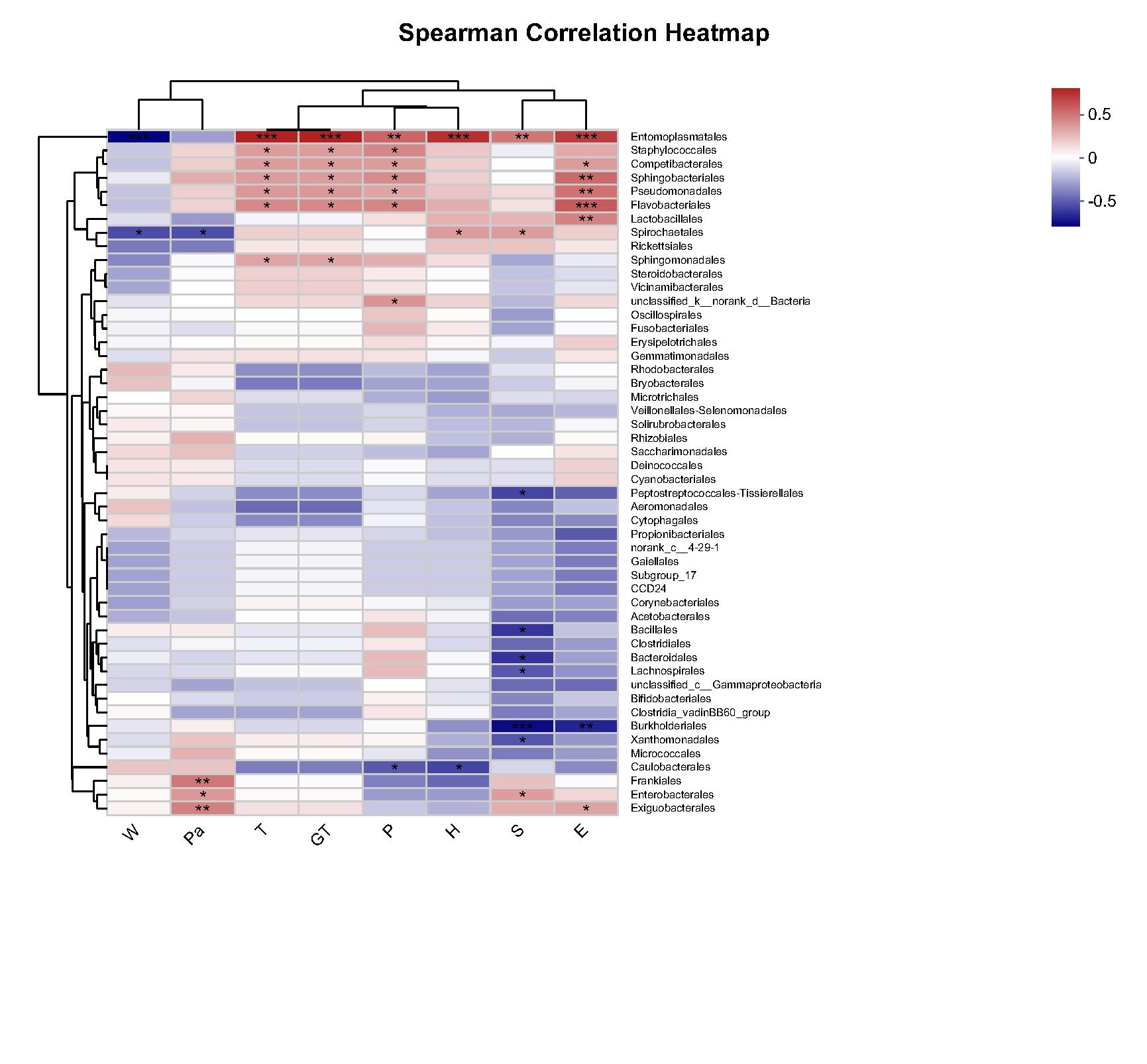


**Supplementary Figure 4.** Geographic Factor Analysis of Gut Microbiota in Different Populations of *C. kiangsu* at the Order Level.


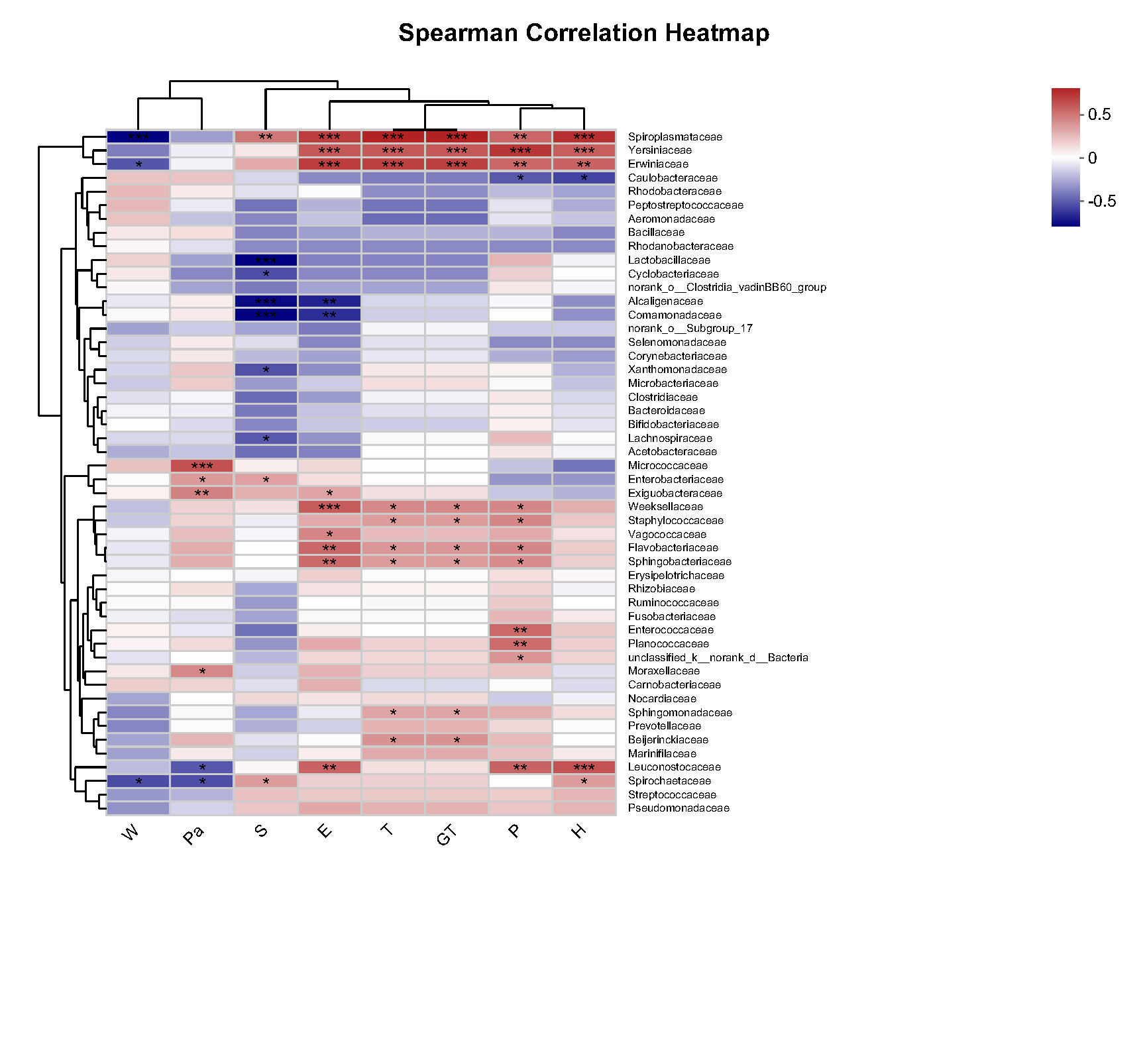


**Supplementary Figure 5.** Geographic Factor Analysis of Gut Microbiota in Different Populations of *C. kiangsu* at the family Level.

## Supplementary Table

| **Sample\Estimators** | **sobs** | **shannon** | **simpson** | **ace** | **chao** | **coverage** |
| --- | --- | --- | --- | --- | --- | --- |
| CB01 | 30 | 0.981909 | 0.486351 | 78.948551 | 48.2 | 0.999194 |
| CB02 | 23 | 0.249891 | 0.889563 | 34.558442 | 30.5 | 0.999425 |
| CB03 | 29 | 1.034031 | 0.451749 | 48.791992 | 55 | 0.999252 |
| CB04 | 28 | 0.241755 | 0.894815 | 48.831996 | 41 | 0.999194 |
| CB05 | 18 | 0.831867 | 0.612815 | 18.879551 | 18.2 | 0.999885 |
| CZ01 | 27 | 0.300227 | 0.901244 | 29.251815 | 30 | 0.99977 |
| CZ02 | 26 | 0.199684 | 0.940928 | 27.770613 | 32 | 0.99977 |
| CZ03 | 58 | 0.741831 | 0.699924 | 66.088716 | 69 | 0.99931 |
| CZ04 | 28 | 1.059226 | 0.427174 | 29.41949 | 28.6 | 0.999827 |
| CZ05 | 25 | 0.214619 | 0.936489 | 25.872237 | 25.5 | 0.999885 |
| GD01 | 93 | 1.996378 | 0.197522 | 97.109293 | 95.4 | 0.999482 |
| GD02 | 39 | 1.569242 | 0.306402 | 47.723447 | 45 | 0.999482 |
| GD03 | 38 | 1.615869 | 0.265542 | 40.654715 | 39.666667 | 0.999712 |
| GD04 | 57 | 1.742097 | 0.23324 | 66.05232 | 65.25 | 0.99931 |
| GD05 | 124 | 2.325539 | 0.170065 | 137.508294 | 138 | 0.998792 |
| GX01 | 42 | 1.275022 | 0.433537 | 43.651669 | 43 | 0.99977 |
| GX02 | 19 | 0.744783 | 0.653825 | 37.363169 | 24 | 0.999655 |
| GX03 | 42 | 1.245479 | 0.448991 | 45.118458 | 43.875 | 0.999655 |
| GX04 | 37 | 1.347625 | 0.417554 | 37.524527 | 37 | 0.999942 |
| GX05 | 17 | 0.72979 | 0.662324 | 17.9 | 17.2 | 0.999885 |
| YN01 | 21 | 1.285755 | 0.401543 | 23.202797 | 22 | 0.999827 |
| YN02 | 14 | 1.402813 | 0.305442 | 62.913223 | 20 | 0.99977 |
| YN03 | 25 | 1.346167 | 0.327126 | 40.262917 | 30.25 | 0.999597 |
| YN04 | 14 | 0.758743 | 0.514631 | 20.048237 | 17.333333 | 0.999712 |
| YN05 | 32 | 0.79075 | 0.544824 | 52.320512 | 37 | 0.999425 |
| YY01 | 42 | 0.954429 | 0.474569 | 50.495413 | 47.5 | 0.999367 |
| YY02 | 6 | 0.009731 | 0.997815 | 22.125 | 9 | 0.999827 |
| YY03 | 4 | 0.007582 | 0.998275 | 5.111111 | 4 | 0.999942 |
| YY04 | 42 | 1.069687 | 0.547008 | 44.52005 | 43 | 0.99977 |
| YY05 | 47 | 0.367869 | 0.893627 | 49.67816 | 52 | 0.999655 |

**Supplementary Table 1.** Relative abundance of gut bacterial communities in C. kiangsu from different geographical populations at the phylum levels. Each color represents one taxon in the corresponding chart. “Others” indicates taxa not listed at the respective taxonomic level.
